# Effect of a Change in the CaCl_2_/Pectin Mass Ratio on the Particle Size, Rheology and Physical Stability of Lemon Essential Oil/W Emulgels

**DOI:** 10.3390/foods12061137

**Published:** 2023-03-08

**Authors:** José Muñoz, Paula Prieto-Vargas, Mᵃ Carmen García, María-Carmen Alfaro-Rodríguez

**Affiliations:** Departamento de Ingeniería Química, Escuela Politécnica Superior, Universidad de Sevilla, C/Virgen de Africa 7, 41011 Sevilla, Spain

**Keywords:** emulgel, low-methoxyl citrus peel pectin, lemon essential oil, sheared gel, rheology, physical stability

## Abstract

A three-step (rotor-stator-microfluidization-rotor stator) protocol was used to prepare 15% lemon essential oil in water emulgels using a mixture of Tween 80 and Span 20 surfactants as low molecular mass emulsifiers and 0.4% low-methoxyl citrus peel pectin as a gelling agent. Ca^2+^ was used as a gel-promoting agent. Different CaCl_2_/pectin mass ratio values from 0.3 to 0.7 were used. Emulgels showed a microstructure consisting of oil droplets embedded in a sheared gel matrix, as demonstrated by bright field optical microscopy. Laser diffraction tests showed multimodal particle size distributions due to the coexistence of oil droplets and gel-like particles. Multiple light scattering tests revealed that the physical stability of emulgels was longer as the CaCl_2_/pectin mass ratio decreased and that different destabilization mechanisms took place. Thus, incipient syneresis became more important with increasing CaCl_2_ concentration, but a parallel creaming mechanism was detected for CaCl_2_/pectin mass ratio values above 0.5. Dynamic viscoelastic and steady shear flow properties of the emulgels with the lowest and highest CaCl_2_/pectin mass ratio values were compared as a function of aging time. The lowest ratio yielded an emulgel with enhanced connectivity among fluid units as indicated by its wider linear viscoelastic region, higher storage modulus, loss modulus and viscosity values, and more shear thinning properties than those of the emulgel formulated with the highest CaCl_2_/pectin mass ratio. The evolution of the dynamic viscoelastic properties with aging time was consistent with the information provided by monitoring scans of backscattering as a function of sample height.

## 1. Introduction

An emulgel can be defined as a complex colloidal gelled structure that involves the entrapment of oil droplets within a gel matrix [1]. These composite systems can generate emulsion-filled gels and emulsion-particulate gels [2]. In the former case, depending on the interactions between droplets and the gel network, droplets can be classified as active fillers, if particles are bound to the gel network, and inactive fillers, if they do not interact or interact weakly with the gel matrix [3]. Emulgels have the advantages of emulsions and hydrogels, with emerging applications such as (a) encapsulation and controlled release of drugs, active ingredients, or bioactive substances, (b) design and modification of food texture, and (c) reduction of fat content. Therefore, today their use is rising and there is an increasing interest in industries as varied as cosmetics, pharmaceuticals, agrochemicals, cleaning products and food [4,5] and are subject to study in numerous scientific articles, such as those by [6,7,8,9,10].

The main components in the formulation of an emulgel are the aqueous solvent, the oil, the emulsifier, and the gelling agent [11]. The selection of the oil phase, and its relative amount, will depend on the final use of the product. Plant-derived oils are increasingly used, not only for pharmaceutical and cosmetic applications but also for food and agrochemical products. Lemon essential oil is a good choice due to its interesting antimicrobial, antifungal, anti-inflammatory, and antioxidant properties [12,13,14,15,16,17,18]. The antioxidant activity of lemon essential oil makes it a very important ingredient in food preservation as a substitute for synthetic preservatives [19]. In addition, it is worth emphasizing that lemon essential oil is considered by the FDA to be a safe ingredient for use as a preservative and flavoring agent [20].

It is well known that the addition of emulsifiers is necessary to decrease the interfacial tension during the formation of emulsions, which involves a reduction of the energy input required. Furthermore, emulsifiers provide some physical stability at least for the short term. Non-ionic surfactants such as Tween 80 and Span 20 are widely used as emulsifiers for the food and cosmetic industry. Tween 80 is a polyoxyethylene (20) sorbitan monooleate, whose hydrophilic-lipophilic (HLB) number is 15. Span 20 is a sorbitan monolaurate, which has an HLB number of 8.6. These surfactants exhibit very low toxicity and eco-friendly chemistry [21,22]. In this work, we use a mixture of both surfactants since a blend of two emulsifiers has been proven to have a synergistic effect, providing more emulsifying efficiency than a simple one [23,24] and allowing a target intermediate HLB (between 8.6 and 15) to be achieved.

Regarding the gelling agent, we can find in the literature works related to the formation of emulgels from (a) proteins (whey protein isolate, gelatin, soy protein isolate, etc.) [2,25,26] (b) polysaccharides (gellan, inulin, k-carragenan, alginate, agar, etc) [27,28,29] and (c) dietary fibers [10]. Different methods have been used to prepare emulgels depending on the gelling agent used: (a) heat treatment, acidification, and cold induction by the addition of salts and enzyme treatment for proteins [6], (b) homogenization and the addition of ions for polysaccharides [7,30] and (c) mechanical activation (e.g., rotor-stator homogenization) for dietary fibers [10].

Nevertheless, just a few studies use pectin as a gel-like matrix to obtain an emulgel. Among the scarce scientific information available it is worth mentioning that Lupi et al. (2015) [31] investigated the complex modulus of emulgels formulated with olive oil, low-methoxyl pectins of different methoxylation degrees and a polyoxyethylene (20) sorbitan monostearate (Tween 60, HLB: 14.9), using a fixed concentration of calcium chloride dihydrate to induce gelling. The authors proposed an empirical predictive model to relate the complex modulus to the oil fraction and pectin concentration. In another study, Hou et al. (2016) [32] fabricated emulgels based on a mix of soy protein and sugar beet pectin through an enzymatic gelation process and studied the influence of the emulsifier and the emulsification process on the microstructure, texture, breakdown properties, and aroma release behavior of the resulting emulsion gels. They found that the emulsification by protein isolate/sugar beet pectin complex resulted in a more compact interfacial network but the release rate of aroma compounds depended on the nature of this. A similar system was studied by Feng et al. (2019) [33] where the emulsion was formulated with Tween 20 (HLB: 16.7) as an emulsifier and gels were obtained by laccase-induced cross-linking reaction and heat treatment. In this work it is concluded that the concentration of emulsion significantly influenced the degree of crosslinking and gel hardness, being a 10 wt% emulsion concentration that provides the desired gelation process and mechanical properties. Recently, Jiang et al. (2021) [34] constructed emulsion gels with low and high-methoxyl pectin, where gelation was induced by acid in an ethanol system. The results revealed that high-methoxyl pectin, with higher molecular weight and lower zeta potential than low-methoxyl pectin, provides a compact structure of gel which is stable during the freeze-thaw cycle. Zhang et al. (2022) [35] studied the fabrication of pectin-based emulsion gels enriched with rhamnogalacturonan-I for protection and sustained release of curcumin. An increase in pectin improved the gel strength and, therefore, enhancing the protection of curcumin against heat-induced degradation. Pectins are polysaccharides conformed of an α-(1–4)-D-galacturonic acid chain, interrupted by 1,2-linked rhamnose units [36]. They are typically used in food products as a thickening agent (viscosity enhancer) or gelling agent although, it has been claimed that they also can function as emulsifiers [37,38]. When the percentage of carboxyl groups, esterified with methanol, is less than 50, they are called low methoxyl pectins (LMP). In LMP, the gel is formed when dissociated carboxyl groups, of different polymer chains, form junction zones according to the shifted egg-box model through divalent-ions bridges such as Ca^2+^ [39]. Other types of interaction are also possible, such as hydrophobic interactions between methoxyl ester groups and hydrophilic interactions between undissociated carboxyl groups and hydroxyl groups via hydrogen bonds [40]. In addition, it is interesting to note that the gel strength reaches an optimal value at intermediate calcium concentrations [41].

In this work, emulgels based on lemon essential oil and low-methoxyl citrus peel pectin will be produced by microfluidization, and by calcium-induced gelation, at room temperature. The influence of the CaCl_2_/pectin mass ratio on the physical stability and rheology of the emulgels obtained will be assessed. The most innovative aspects of this research are (a) to use of a microfluidized emulsion for the preparation of a pectin-based emulgel since most previous works were carried out by using rotor-stator or high-pressure valve homogenizers [31,33] and (b) to explore the effect of aging effects on the microstructure and rheology of emulgels based on a sheared gel matrix. This study will provide useful progress in the knowledge and development of pectin-based emulgels. It must be emphasized that this research is a contribution to the circular economy of lemon juice production since further applications are explored for lemon peels. These are mainly used as a raw material of the pectin used in industrial subproducts, and also for lemon essential oil.

## 2. Materials and Methods

### 2.1. Materials

Emulgels were formulated using lemon essential oil purchased from Bidah-Chaumel (Spain), Tween 80 (HLB 15) and Span 20 (HLB 8.6), both from Sigma Aldrich (Spain), low-methoxyl citrus pectin (Aglupectin LC S18YP, methoxylation degree: 37–41%), kindly supplied by JRS Silvateam Ingredients Srl (Italy), citrate buffer solution containing deionized water, citric acid and tris-sodium citrate 2-hydrate at pH = 4.2, both reagents supplied by Panreac (Spain), calcium chloride dehydrate provided by Sigma-Aldrich (Spain) and, deionized water.

The emulgels studied contained 15 wt% lemon essential oil, 15 wt% of a mixture of Tween 80 and Span 20, (Tween 80/Span 20) mass ratio: 1.13 which results in an HLB number of 12, which had previously been optimized. The present study was carried out with a fixed pectin concentration (0.4 wt%) and a variable CaCl_2_/pectin mass ratio (R) which ranged from 0.3 to 0.7. These values were previously selected to obtain samples which looked homogeneous by visual observation.

Concerning the protocol of emulgel preparation, firstly, a coarse emulsion (batches of 200 g) was prepared, at room temperature, using a rotor-stator Ultra-Turrax T50 homogenizer with the S50-G45F dispersion unit. The aqueous phase was prepared by adding the appropriate amount of deionized water, emulsifiers (Tween 80 and Span 20), citrate buffer solution (pH = 4), and pectin while stirring for 3600 s at 2000 rpm. Afterwards, the lemon essential oil was gradually added to the aqueous phase stirring at 2000 rpm for 40 s, and subsequently, the same device was used for 90 additional seconds at 4000 rpm. Subsequently, finer emulsions were obtained using a microfluidizer M-110P (Microfluidics, EEUU) with chambers F12Y and H30Z arranged in series, after two passes at 137.9 MPa. Finally, emulgels were obtained by adding CaCl_2_ in the form of a solution (7% w/v stock solution) and stirring at 8000 rpm for 30 s in the Ultra-Turrax T50 homogenizer. This final mechanical treatment followed the method reported by Lupi et al. (2015) [31] and Lorenzo et al. (2013) [7]. Once prepared, the emulgels were left to rest in an oven at 25 °C until characterization.

In Table 1 the composition of the emulgels is shown. The RX nomenclature will be used to denote the emulgels, with X being the CaCl_2_/pectin mass ratio; e.g., R0.3 standing for an emulgel formulated with a CaCl_2_/pectin mass ratio of 0.3.

### 2.2. Sample Characterization

#### 2.2.1. Particle Size Distribution

The particle size distribution (PSD) of samples was obtained using a Malvern Mastersizer 2000 laser diffraction device. The indexes of refraction for lemon essential oil and water were 1.474 and 1.330, respectively and the absorption index was 0.001. Each test was performed at room temperature and in duplicate.

#### 2.2.2. Multiple Light Scattering

The physical stability of all the samples studied was characterized by Turbiscan Lab-Expert equipment (Formulation, France) based on the principle of multiple light scattering. The analyzer is equipped with a pulsed near-infrared light source (λ = 880 nm) and synchronous optical detectors that determine the intensity of the backscattered light. In opaque samples, the backscattering profiles are analyzed rather than the transmitted light profiles. Changes in the backscattering profiles, as a function of measuring cell height, can be used to detect and characterize physical instabilities. Thus, local variations of the profiles at the top or bottom of the sample are the consequence of particle migration, whereas global variations along the whole height of the sample are the result of particle size variations.

#### 2.2.3. Rheological Characterization

Small-amplitude oscillatory tests and flow curves were performed using a Haake Mars II controlled stress rheometer (Thermo-Scientific, Karlsruhe, Germany), with a serrated plate-plate geometry, PP60R (diameter: 60 mm and gap: 1 mm). The equilibration time before the rheological measurement was 300 s and the temperature was fixed at 20 °C.

A stress sweep from 0.1 to 20 Pa was carried out at a frequency of 1 Hz to estimate the linear viscoelastic range. Subsequently, mechanical spectra were obtained from 0.008 to 10 Hz at a shear stress amplitude within the linear viscoelastic zone. Flow curves were run using a controlled stress protocol from 0.1 to 10 Pa. All measurements were made in duplicate with fresh samples.

The flow curves were fitted to the power-law equation:

η_a_ = K·γ ˙ ^(n−1)^ (1)

where η_a_ is the apparent viscosity (Pa·s), γ ˙ the shear rate (s^−1^), K is the consistency index (Pa·s^n^), and n is the so-called “power-law flow index”.

#### 2.2.4. Optical Microscopy

Emulgels were observed using an optical microscope, Axio Scope A1 (Carl Zeiss). Samples were observed at room temperature, using 100× oil immersion objective using the bright field technique.

#### 2.2.5. Statistical Analysis

The standard deviation of the mean of replicates was calculated to determine the occurrence of significant differences among the results obtained.

## 3. Results and Discussion

### 3.1. Particle Size Distribution

**Figure 1:**
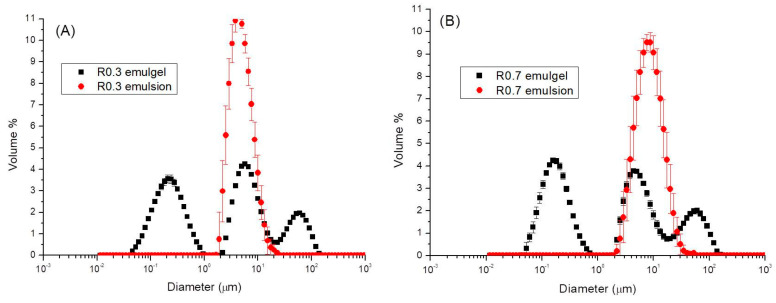
Comparison of particle size distributions for emulgels (black symbol) with (**A**) a CaCl_2_/pectin mass ratio of 0.3 (R0.3), (**B**) a CaCl_2_/pectin mass ratio of 0.7 (R0.7) and the corresponding fresh emulsions without CaCl_2_ prepared by microfluidization (red symbol). Bars correspond to standard deviation. Tests were done at room temperature.

Figure 1A,B shows a comparison of the PSDs of emulgels to those of the corresponding fresh emulsions containing pectin, previously produced by microfluidization (M110P microfluidizer) before adding calcium chloride. Results for the lowest (0.3) and highest (0.7) R values studied are presented by way of example. It can be observed that emulsions produced by microfluidization, without CaCl_2_, exhibited a monomodal distribution whose population peak is located at around 5–8 μm, which is consistent with the occurrence of depletion flocculation of oil droplets due to the presence of pectin in the continuous phase. If these PSDs are compared with those of the corresponding emulgels, the latter exhibit a multimodal distribution, this being the case regardless of the R value considered. Namely, three main peaks can be clearly observed. Emulgels exhibit a first peak located at around (180–200) nm and a third peak located around 60 μm. The comparison of PSDs of emulsions and emulgels seems to indicate that the first and third population peaks, appearing for emulgels, are consistent with the occurrence of a lemon essential oil emulsion dispersed in a sheared gel, consisting of nanogels, microgels and gelled-particles of pectin. Sheared or fluid gels are formed when the gelation process is to some extent interrupted by shear forces such that the aggregation of macromolecules is prone to form a strong or true gel, hindered by hydrodynamic forces and gel-like clusters are partially disrupted [42]. These fluid gels exhibit interesting rheological properties upon the onset of shear flow and viscoelastic properties with a predominance of the elastic component over the viscous one [43,44].

The multimodal PSDs exhibited by the emulgels prepared, which consist of a dispersion of an emulsion into sheared gels, prevent the analysis of results provided by the laser diffraction technique from being made based on mean diameters and any parameter describing the width of the distribution.

### 3.2. Physical Stability

**Figure 2:**
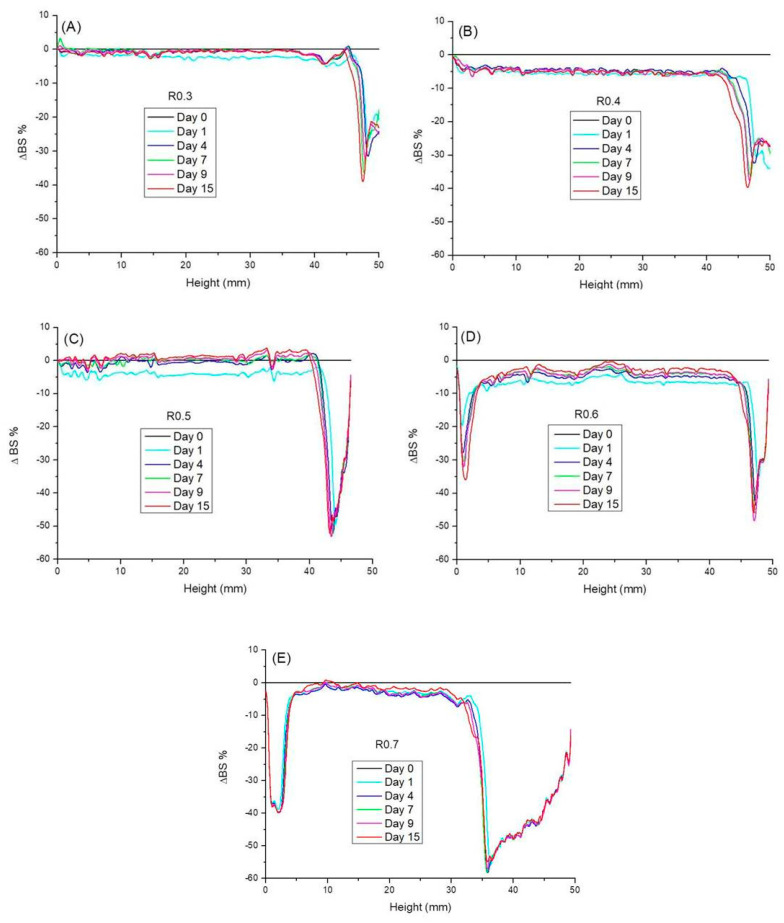
Delta Backscattering (ΔBS%) as a function of the height of the sample and aging time for emulgels with a CaCl_2_/pectin mass ratio of (**A**) 0.3, (**B**) 0.4, (**C**) 0.5, (**D**) 0.6 and (**E**) 0.7. Room temperature.

**Figure 3:**
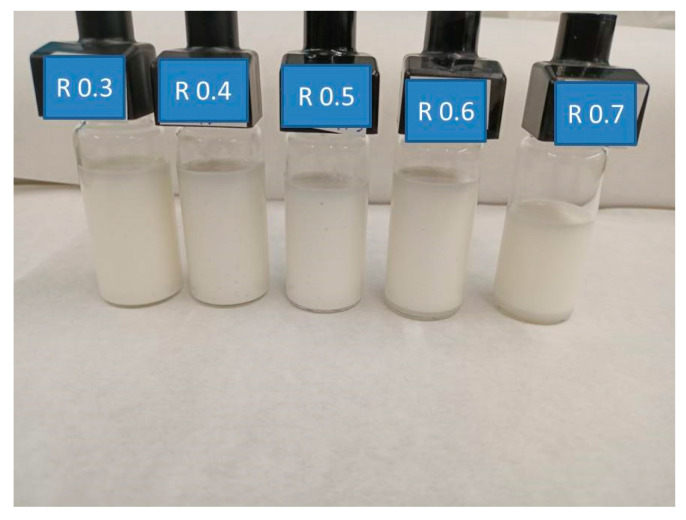
Photography provides of the emulgels in the measuring cells of Turbiscan, two months after preparation, as a function of the CaCl_2_/pectin mass ratio, R.

The physical stability of the emulgels was studied using the multiple light scattering (MLS) technique to identify instability phenomena at short times because this technique is capable of detecting small changes before a change on a macroscopic scale can be detected by the naked eye. It should be emphasized that it is not necessary to dilute the samples to use this technique; hence it is a non-intrusive method. In addition, this technique does not require providing physical parameters of either the sample or its components, like the refraction index, absorption index or density. Figure 2 shows ΔBS% profiles along the whole height of the measuring cell as a function of the aging time of the emulgels studied. The ΔBS% is defined as (BSt − BSt = 0) where BSt is the % of backscattering at a given time (t) and BSt = 0 is the initial backscattering. According to the principle reported by Mengual et al. (1999) [45], a decrease or increase in backscattering at the bottom of the measuring cell is related to the occurrence of a creaming or sedimentation, respectively. Changes in the middle of the cell are due to an increase of particle size without distinguishing between flocculation, coalescence, or Ostwald ripening, and changes in backscattering at the top can be due to different causes, such as coalescence, oiling off or decrease in the sample volume. As can be seen in Figure 2A, the (ΔBS%) profile for R0.3 emulgel is close to zero along most of the sample height, supporting that changes in particle size did not occur in 15 days. However, a clear decrease in ΔBS% was observed on the top part of the sample from day one of aging time. This effect was more important and took place at progressively lower sample height as aging time evolved. The R0.4 emulgel exhibited just a small decrease in ΔBS% (−5%) along most of the sample height if compared with the initial scan (Figure 2A). However, this change is not significant and cannot be interpreted as indicative of a change in particle size as supported by Celia et al. (2009) [46]. These authors claimed that no variation in particle size occurs when the backscattering profile is within the interval ±2%. Changes greater than 10%, either positive or negative, would be associated with an unstable formulation. The rest of the emulgels studied did not show changes in ΔBS% values higher than 10% at intermediate sample height (Figure 2C–E). In fact, the ΔBS% values were ≤5% for emulgels aged for 15 days, supporting the lack of particle size changes. Regarding variations of ΔBS% values in the upper part of samples, these were progressively more important and took place at a lower and lower height as the CaCl_2_/pectin mass ratio was increased. This was the first indication of the occurrence of incipient syneresis from the first aging day, although it could not be visually observable in the measuring cells (Figure 3), until a much longer aging time (2 months) than the time scale of the multiple light scattering tests done. In addition, the emulgels with CaCl_2_/pectin mass ratio ≥0.6 also underwent destabilization by creaming as demonstrated by the decreasing values of ΔBS% at the bottom of the samples.

**Figure 4:**
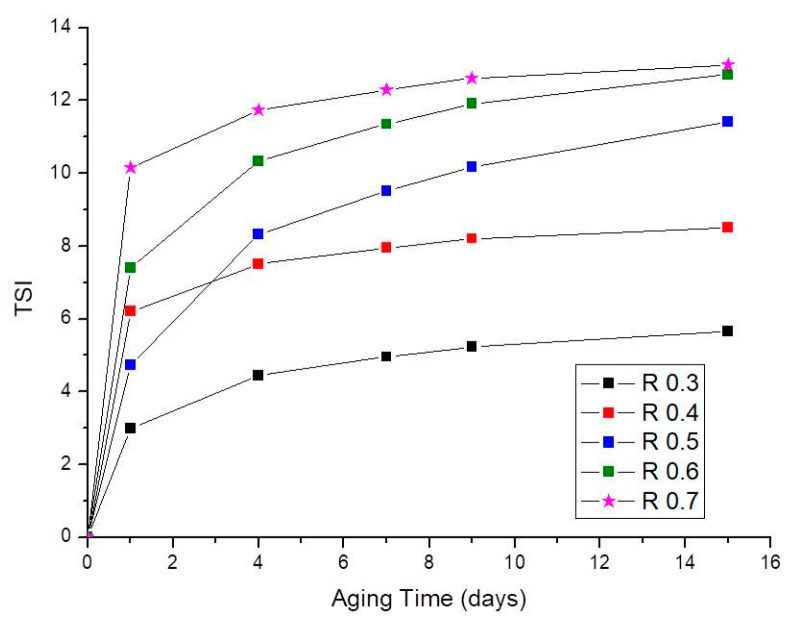
Turbiscan Stability Index was calculated for the entire height of the measuring cell with aging time for emulgels as a function of the CaCl_2_/pectin mass ratio.

It is also possible to compare the physical stability of emulgels at a given time (15 days), by calculating the Turbiscan stability index (TSI) [47]. It should be noted that the Turbiscan Stability Index (TSI) is a very sensitive parameter to monitor destabilization phenomena of dispersion since it is calculated by adding the variations in backscattering values from every scan to the following one. A high TSI value means that there are many changes when comparing the different scans; hence it is associated with extensive migration of particles. Alternatively, it can be associated with simultaneous or independent increases in particle size, oiling off or solvent expulsion from the sample matrix. A low TSI value means the opposite; i.e., the sample is quite homogeneous and stable against phase separation. Given that various destabilization mechanisms were simultaneously involved in the stability of the emulgels studied, the TSI was calculated taking into account the whole height of the sample. Figure 4, shows that the TSI increased with aging time and CaCl_2_/pectin mass ratio. The destabilization rate went down with aging time as demonstrated by the decreasing slopes of the curves; i.e., the main changes in backscattering took place at a short aging time. The evolution of this parameter supports that the emulgel with the lowest CaCl_2_/pectin mass ratio studied (R0.3) was the most stable, while the worst stability was shown by the emulgel with the highest calcium content (R0.7), congruently with the evolution observed for ΔBS% (Figure 2E).

### 3.3. Rheological Characterization

In this section, the viscoelastic properties and flow behavior of the emulgels with the best and worst physical stability are compared as a function of aging time.

**Figure 5:**
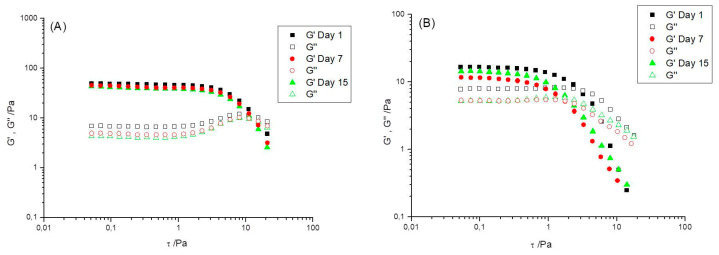
Stress sweeps as a function of the aging time for emulgels with a CaCl_2_/pectin mass ratio of (**A**) 0.3 and (**B**) 0.7. T = 20 °C.

Figure 5 shows the storage, G′, and loss, G″, moduli versus stress amplitude, *τ*, at 1 Hz to estimate the linear viscoelastic region (LVR) of R0.3 and R0.7 samples as a function of aging time. The storage modulus, G′, at 1 Hz; i.e., the elastic component turned out to be greater than the corresponding loss modulus, G″; i.e., the viscous component for both emulgels. The critical shear stress amplitude, limiting a linear response, where the sample microstructure is not affected by shear, was around 1 Pa for R0.3 emulgel (Figure 5A). The initial growth of G″ above the shear stress amplitude range, showing nearly constant G′ and G″ values, allowed the onset of nonlinear viscoelastic behavior to be accurately located and is a consequence of a viscous dissipation phenomenon associated with some shear-induced rearrangements in the contact zones of gel-like domains coexisting with embedded droplets in the emulgels studied (see Section 3.4). A similar interpretation was given by Alfaro et al. (2000) [48] when describing the same phenomenon for surfactant lamellar liquid crystals. It should be noted that the critical shear stress amplitude locating the end of the linear viscoelastic range has been associated with the degree of association of structural units forming material or the number and lifetime of semipermanent entanglements among macromolecules [49].

The extension of the linear viscoelastic range turned out to be larger for R0.3 emulgel than for R0.7 (Figure 5A vs. Figure 5B). The critical stress amplitude limiting the linear viscoelastic response of the latter was 0.3 Pa, as better detected by the fall of G′ values. These results support the fact that the network among gel-like domains and flocs of droplets is stronger, more compact and more structured for the emulgel with the lowest CaCl_2_/pectin mass ratio studied (R0.3), than for the emulgel formulated with an excess of calcium cations (R0.7). This interpretation is further supported by the facts that (a) G′ values at 1 Hz are higher for the R0.3 emulgels than for R0.7 (the loss tangent values would be lower for R0.3, indicating a more elastic response) and (b) the dynamic viscoelastic functions of the R0.3 emulgel are more stable as a function of aging, congruently with the multiple light scattering results obtained with the Turbiscan Lab-Expert used.

**Figure 6:**
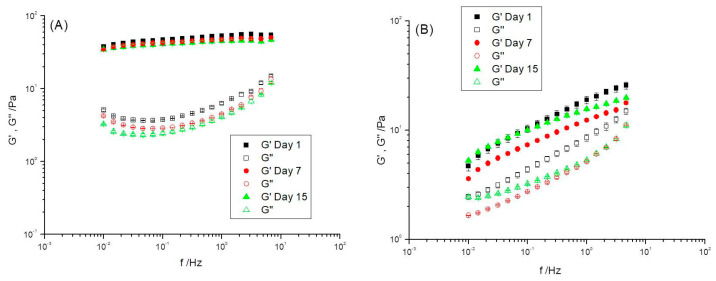
Influence of frequency on storage modulus, G′, and loss modulus, G″ for emulgels with a CaCl_2_/pectin mass ratio of (**A**) 0.3 and (**B**) 0.7. The standard deviation is also plotted. T = 20 °C.

The mechanical spectrum of R0.3 (Figure 6A) is consistent with the formation of a solid-like weak gel-like structure with G′ values above those of G″ in the whole frequency range studied, and a slight dependence on frequency. This behavior is typical of different types of structured materials, e.g., concentrated emulsions and suspensions, suspo-emulsions, synthetic polymers, biopolymer-based and protein-based weak gels, etc. An aging time of 15 days resulted in a small decrease in G′, which was not significant from a statistical point of view, as illustrated by the standard deviation bars. However, this effect was slightly clearer for G″. These results agree with the weak destabilization process of the emulgel formulated with a 0.3 CaCl_2_/pectin mass ratio monitored by backscattering profiles (Figure 2A).

The mechanical spectrum of R0.7 emulgel, like R0.3, is typical of a weak gel-like structure but with a dramatic fall of G′ values and a higher frequency dependence (Figure 6B). This would imply a lower level of connectivity among structural units responsible for the shear flow, i.e., the results obtained are consistent with the existence of a weaker network structure as pointed out in the pioneering paper by Gabriele et al. (2001) [50] on food weak gels.

As far as the aging effects on the mechanical spectra of emulgel R0.7 are concerned, a clear tendency could not be observed due probably to the simultaneous influence of different destabilization phenomena (creaming and incipient syneresis) as supported by backscattering data shown in Figure 2E. G′ and G″ data obtained for each individual mechanical spectrum had excellent repeatability as indicated by the very small standard deviation bars shown in Figure 6B. Therefore, the different mechanical spectra obtained for R0.7 emulgel did show real “snap-shots” of the evolving viscoelastic responses of the emulgel as its heterogeneous microstructure changed with aging time.

**Figure 7:**
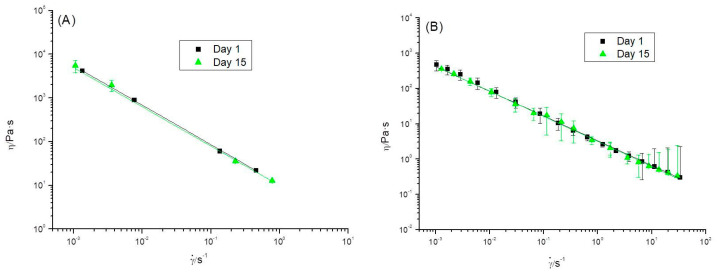
Steady shear flow curves for emulgels with a CaCl_2_/pectin mass ratio of (**A**) 0.3 and (**B**) 0.7, as a function of aging time. Standard deviation is also plotted. Continuous Lines correspond to the power-law model fit T = 20 °C.

The steady shear flow behavior of the emulgels studied was shear thinning (Figure 7) since the steady-state apparent viscosity decreased with the shear rate. The results obtained fitted fairly well with the power-law equation.

Regardless of aging time, Figure 7 shows that viscosities exhibited by the R0.3 emulgel are one order of magnitude higher than the emulgel formulated with the highest CaCl_2_/pectin mass ratio (R0.7). The fitting parameters to the power-law equation are shown in Table 2. The consistency index values exhibited by R0.3 emulgel were clearly higher than those of R0.7 emulgel, congruently with its stronger structure at a quiescent state as indicated by its mechanical spectrum. Likewise, the flow index n is lower for the R0.3 emulgel indicating that this emulgel has more pronounced shear-thinning properties. It is important to point out that both emulgels exhibited rather stable flow curves as a function of aging time (at least in the time period covered by this study: 15 days).

### 3.4. Optical Microscopy Study

**Figure 8:**
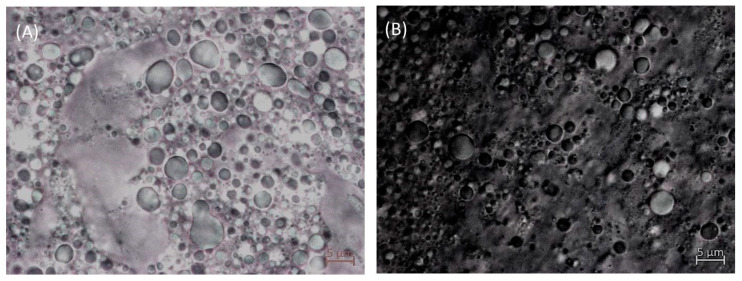
Microphotograph for 24 h aged emulgels with a CaCl_2_/pectin mass ratio of (**A**) 0.3 and (**B**) 0.7. 100× oil immersion objective lens.

Figure 8A,B show the microstructure of the R0.3 and R0.7 emulgels at room temperature observed by optical microscopy using a bright field and an oil immersion objective of 100×. Both micrographs revealed that the emulgels studied consisted of a complex dispersion of lemon essential oil droplets embedded in a matrix formed by a Ca^2+^ induced-sheared gel. Particle gels present a wide range of sizes in agreement with the multimodal particle distribution determined by laser diffraction (Figure 1). No significant differences could be observed by analyzing micrographs of R0.3 and R0.7 in several fields of observation. Given that the micrographs were obtained challenging the resolution of optical microscopy, a deeper insight into the complex microstructure of these emulgels will only be achieved by using a methodology based on electron microscopy. Selecting the specific methodology (cryo-SEM, FESEM, TEM) will be one of the tasks to be tackled when undertaking future studies on these materials.

An overall analysis of the results obtained suggests that the properties of the emulgels prepared, strongly depended on the method of preparation. In reality, the emulgels prepared consisted of a dispersion of oil droplets embedded in a matrix made of a sheared gel with a wide range of particle gel sizes. This complex microstructure was achieved by using a preparation protocol in series. A primary O/W emulsion was first prepared by rotor-stator homogenization, which subsequently was treated by microfluidization to obtain the so-called secondary or final emulsion. Immediately a concentrated solution of CaCl_2_ was added and mixed with the secondary emulsion by rotor-stator technology to promote gelation of the low-methoxyl pectin used. However, the gel formation was partially interrupted by shear and extensional forces associated with the final homogenization step. This phenomenon has been well documented when describing the formation of fluid gels based on gellan gum [42,43,44] and other polysaccharides able to form strong gels, like alginates [51]. In this work, the effect of shear when mixing the secondary emulsion and the CaCl_2_ solution must be more efficient in disrupting the incipient gel formation in the system containing the lowest concentration of Ca^2+^ cations, resulting in the formation of a high number of small particle gels. This, in turn, may favor interparticle interactions and connectivity in the emulgel formulated with the lowest CaCl_2_/pectin mass ratio (0.3), confirming that this emulgel exhibited the best results concerning physical stability, viscoelasticity and steady shear flow properties (high viscosity and better shear thinning behavior).

## 4. Conclusions

Emulgels of lemon essential oil containing low-methoxyl pectin could be prepared by using series rotor-stator, microfluidization and rotor-stator devices. Optical microscopy micrographs confirmed that the emulgels produced consisted of lemon essential oil/water emulsions embedded in a sheared gel induced by the addition of CaCl_2_. The concentration of Ca^2+^ did markedly influence the physical stability of the emulgels prepared. All emulgels were prone to undergo syneresis. However, this phenomenon was faster as the CaCl_2_ concentration increased.

The Emulgels produced exhibited viscoelastic behavior with a predominant elastic component, over viscous and shear thinning flow behavior. The emulgel formulated with the lowest CaCl_2_/pectin mass ratio (R0.3) presented a more compact and stronger structural network than the emulgel formulated with an excessive CaCl_2_ concentration, according to the rheological results obtained.

It can be concluded that the best formulation was R0.3 considering its physical stability and its rheological properties.

Further experimental work must be carried out to search for lemon essential oil emulgels with enhanced physical stability by studying the effect of CaCl_2_/pectin ratio below the lowest value covered by the present study, and by using a low-energy mixer in the final step of the preparation process. The contribution of the sheared gel matrix to the rheology of these emulgels will be studied by carrying out the same preparation protocol without adding the oil phase. In addition, the effect of storage temperature and measuring temperature on the rheology and stability of these systems should be studied on account of their importance in assessing their potential applications. Last but not least, electron microscopy techniques must be used to gain a deeper insight into the microstructure of the sheared gel matrix embedded by oil droplets of these emulgels.

## Figures and Tables

**Table 1:** Composition of the emulgels studied. RX stands for the CaCl_2_/pectin mass ratio.

wt%	R0.3	R0.4	R0.5	R0.6	R0.7
Pectin	0.4	0.4	0.4	0.4	0.4
CaCl_2_	0.12	0.16	0.20	0.24	0.28
Tween 80	7.95	7.95	7.95	7.95	7.95
Span 20	7.05	7.05	7.05	7.05	7.05
Lemon Oil	15	15	15	15	15
Water	69.48	69.44	69.40	69.36	69.32

**Table 2:** Fitting parameters of the power-law equation for the shear rate dependence of steady shear apparent viscosity values of emulgels R0.3 and R0.7. Temperature: 20 °C.

	Day	K (Pa·s^n^)	n	R2
R 0.3	1	10.65 ± 0.41	0.10 ± 0.01	0.991
	15	9.96 ± 0.21	0.10 ± 0.00	0.992
R 0.7	1	3.31 ± 0.07	0.30 ± 0.01	0.985
	15	3.17 ± 0.07	0.29 ± 0.00	0.986

## Data Availability

The data presented in this study are available on request from the corresponding author.
